# The Mechanism of Inflammatory Factors and Soluble Vascular Cell Adhesion Molecule-1 Regulated by Nuclear Transcription Factor NF-*κ*B in Unstable Angina Pectoris

**DOI:** 10.1155/2022/6137219

**Published:** 2022-07-30

**Authors:** Qingfeng Su, Linhu Zhang, Zhenhui Qi, Bo Huang

**Affiliations:** ^1^Department of Cardiology, Shanxi Provincial People's Hospital, Taiyuan, Shanxi, China; ^2^Laboratory Animal Center, Shanxi Provincial People's Hospital, Taiyuan, Shanxi, China

## Abstract

This work is aimed at exploring the mechanism of inflammatory factors and soluble vascular cell adhesion molecule-1 (sVCAM-1) regulated by nuclear transcription factor-*κ*B (NF-*κ*B) in unstable angina pectoris (UAP). 60 patients with unstable angina pectoris (UAP), 60 patients with stable angina pectoris (SAP), and some healthy people (controls) were selected and included. Peripheral venous blood (PVB) of all subjects was collected to detect blood routine. The enzyme-linked immunosorbent assay (ELISA) was adopted for detecting Visfatin, sVCAM-1, soluble intervascular cell adhesion molecule-1 (sICAM-1), and inflammatory factors; flow cytometry (FCM) was to detect the CD63 and CD62P; real-time fluorescence quantitative polymerase chain reaction (rt-qPCR) was employed to detect the NF-*κ*B1, NF-*κ*B2, and REL mRNA. The hs-CRP results of UAP group, SAP group, and control group were 11.12 ± 1.5 mg/L, 10.23 ± 1.3 mg/L, and 4.51 ± 1.1 mg/L, respectively. The CD62P results of UAP group, SAP group, and control group were 16.07 ± 2.5%, 11.09 ± 1.8%, and 22.15 ± 0.4%, respectively. The high-sensitivity C-reactive protein (hs-CRP), inflammatory factors (IL-6, IL-17, IL-23, IL-1*β*, and tumor necrosis factor *α* (TNF-*α*)), CD63, CD62P, NF-*κ*B1, NF-*κ*B2, and REL mRNA were obviously higher in the SAP group compared than the indicator values in the control group (*P* < 0.05). The relative REL expression results of UAP group, SAP group, and control group were 3.77 ± 1.5, 2.2 ± 0.6, and 1 ± 0.4, respectively. The inflammatory factors, Visfatin, sVCAM-1, sICAM-1, CD63, CD62P, NF-*κ*B1, NF-*κ*B2, and REL mRNA in the UAP group showed higher levels in contrast to the other two groups (*P* < 0.05). In summary, UAP patients had marked activation of the IL-23/IL-17 inflammatory axis, high expressions of sVCAM-1 and sICAM-1, and activation of the NF-*κ*B pathway. Increase of inflammatory factors and sVCAM-1 regulated by NF-*κ*B was closely correlated with UAP.

## 1. Introduction

Coronary heart disease (CHD), also known as ischemic heart disease (IHD), is stenosis or occlusion of the coronary lumen caused by coronary atherosclerotic plaque, resulting in acute myocardial ischemia and hypoxia [1]. Epidemiological survey results show that CHD has become a major disease that seriously endangers human life and health and shows a trend of getting younger and increasing its incidence year by year [2]. Statistics show that CHD and stroke are the first causes of death in patients, and cardiovascular disease (CVD) mortality ranks first in China [3]. Unstable angina pectoris (UAP) is a serious and common CHD. The sudden rapid enlargement, rupture or surface damage of coronary atherosclerotic plaque, local platelet accumulation and thrombosis, and vasospasm are the pathological basis of UAP [4, 5]. UAP is intermediate between stable angina pectoris (SAP) and acute myocardial infarction (AMI), with a high incidence for progression [6]. At present, the main methods of clinical diagnosis of UAP are the clinical symptoms of patients and the dynamic changes of the electrocardiogram (ECG) during the onset of the disease, but the diagnosis results are prone to be affected by the patient's medical history, so the clinical diagnosis efficiency is low [7].

NF-*κ*B is an active transcription factor present in cardiomyocytes, vascular smooth muscle cells, and endothelial cells and is an inflammatory mediator of myocardial ischemia. The role of NF-*κ*B in activating various inflammatory responses, amplifying inflammatory signals, and inducing the formation of atherosclerotic plaques has been demonstrated. TNF-*α* is an important inflammatory cytokine and is proved to be closely related to the prognosis of UAP [8]. Previous studies have confirmed that some biological markers can also be used in the diagnosis of CHD, such as interleukin (IL), tumor necrosis factor (TNF), and nuclear transcription factor-*κ*B (NF-*κ*B). NF-*κ*B is relevant to the inflammatory response, immune response, apoptosis, and other processes [9]. NF-*κ*B can activate certain cytokines (IL-6, IL-12, IL-1*β*, and TNF-*α*) and then participate in the early immune response and inflammatory response [10]. Inflammatory response of the body is closely relevant to inflammatory cytokines or inflammatory mediator genes, so NF-*κ*B, as a transcription factor, can mediate in the occurrence and development of inflammatory responses. In addition, soluble vascular cell adhesion molecule-1 (sVCAM-1) and soluble intervascular cell adhesion molecule 1 (sICAM-1) are immunoglobulin superfamily members in peripheral blood, which participate in and mediate the vascular endothelial inflammatory injury response caused by adherent vascular endothelial cells [11]. Therefore, they can be used to evaluate the degree of vascular endothelial inflammatory damage.

The specific mechanism of NF-*κ*B-mediated inflammatory response and sVCAM-1 in the process of CHD has not been fully elucidated. To this end, patients with UAP and SAP under CHD were undertaken as subjects, and differences in levels of NF-*κ*B-mediated inflammatory factors and sVCAM-1 of them were compared and analyzed. The results were intended to offer a reference for further exploration of the pathological mechanism of CHD.

## 2. Materials and Methods

### 2.1. Research Subjects

60 UAP patients, 60 SAP patients, and 30 healthy volunteers in our hospital from March 2019 to January 2022 were included and rolled into a UAP group, an SAP group, and a control group, respectively. UAP patients met the criteria given in *Guidelines for The Diagnosis and Treatment of Non-ST Segment Elevation Acute Coronary Syndrome*, and SAP patients met the descriptions mentioned in *Guidelines for The Diagnosis and Treatment of Chronic Stable Angina Pectoris* [12]. Patients in the UAP group had to meet the below items: patients with initial exertional angina pectoris, worsening exertional angina pectoris, and resting angina pectoris; patients with typical symptoms of angina pectoris within 24 hours of admission and no more than 30 minutes of symptom duration; patients whose ECG examination showed symptoms of myocardial ischemia or had old Q-wave myocardial infarction or had a history of coronary artery bypass grafting (CABG), percutaneous transluminal coronary angioplasty (PTCA), and old myocardial infarction (OMI); and patients whose coronary angiography showed at least one epicardial coronary stenosis more than 50%. Patients meeting below criteria can be included in the SAP group: patients with chronic SAP diagnosed by previous coronary angiography. Exclusion criteria for patients in the UAP group and the SAP group were summarized as follows: patients with unclear diagnosis, abnormal liver or kidney function, autoimmune diseases, acute and chronic infectious diseases, malignant tumors, blood system diseases, and other types of heart disease, AMI within the past 30 days, or surgical history or trauma within 30 days before admission. The controls were from the physical examination center of our hospital, and those with CHD were excluded from the clinical examination.

All research subjects, including the SAP patients, UAP patients, and controls, were aware of the research methods and signed the informed consent form, and the research was approved by the medical ethics committee of the hospital.

### 2.2. Physical Examination

Firstly, it should measure the body weight and height of the research subjects and calculate the body mass index (BMI) with [weight (kg)/height (m^2^)]. Secondly, after 5 minutes of resting by sitting and standing quietly, the blood pressure and heart rate were measured with a sphygmomanometer and a stethoscope for 1 minute, respectively. Thirdly, the echocardiogram of the research subjects was detected by using color Doppler ultrasound diagnostic system (IE33, Philips, Netherlands), and the mean value of the left ventricular end-diastolic diameter (LVEDD) in 3 cardiac cycles was calculated and taken. In addition, the left ventricular ejection fraction (LVEF) was calculated accordingly. Finally, a coronary angiography was performed on the research subjects by using the angiography and interventional therapy system (AXIOM Artis U, Siemens, Germany) and then calculate the degree of coronary stenosis by equation: [(normal lumen diameter at the proximal end of the stenosis − lumen diameter of the stenotic vessel)/proximal end of the stenosis normal lumen diameter × 100%].

### 2.3. Biochemical Index Detection by Blood Routine

5 mL of peripheral venous blood (PVB) of the research all subjects was collected on an empty stomach and stored at room temperature to detect the routine biochemical indicators within 2 hours. An automatic biochemical analysis system (AU5800, Beckman Coulter Co., Ltd., USA) was employed for detection of total cholesterol (TC), triglyceride (TG), low-density lipoprotein cholesterol (LDL-C), high-density lipoprotein cholesterol (HDL-C), creatinine (Cre), uric acid (UA), fasting plasma glucose (FPG), and high-sensitivity C-reactive protein (hs-CRP).

### 2.4. Detection of Visfatin, sVCAM-1, and sICAM-1 in the Blood

5 mL PVB was collected from the all subjects on an empty stomach, treated with heparin sodium anticoagulation, and centrifuged at 3500 rpm for 15 min for supernatant collection. Sandwich enzyme-linked immunosorbent assay (ELISA) technology was adopted for detecting the contents of Visfatin, sVCAM-1, and sICAM-1 by referring to the instructions of Aviscera Bioscience ELISA kit. The model of the Visfatin ELISA kit was SK00121-06, the model of the sVCAM-1 ELISA kit was SK00251-01, and the model of the sICAM-1 ELISA kit was SK00250-02.

### 2.5. Detection of CD63 and CD62P in the Blood

5 mL PVB was collected from the all subjects on an empty stomach, treated with heparin sodium anticoagulation, and centrifuged at 3500 rpm for 15 min for supernatant collection. The CD63 and CD62P in the blood were detected by CytoFLEX flow cytometer (FCM) from Beckman Coulter Co., Ltd. The CD63 and DC62P monoclonal antibodies were provided by the British company Abcam.

### 2.6. Detection on Inflammatory Factors in the Blood

Again, 5 mL PVB was collected when the all subjects were on an empty stomach, treated with heparin sodium anticoagulation, and centrifuged with the same conditions and duration with above to keep the supernatant. The sandwich ELISA technology was to detect contents of IL-6, IL-17, IL-23, IL-1*β*, and TNF-*α* in serum by referring to the kit instructions of R&D Systems in the United States by using PD6050, PD1700, PD2300B, PDLB50, and PDTA00D, respectively.

### 2.7. Detection on Levels of NF-*κ*B1, NF-*κ*B2, and REL mRNA

2 mL PVB of research all subjects was taken on an empty stomach. 6 mL of red blood cell lysate was dropped on ice to fully lyse red blood cells, which were centrifuged at 12,000 rpm for 5 min at 4°C. Precipitate was added 1 mL of erythrocyte lysate to lyse the red blood cells and then centrifuged at the same conditions to remove the supernatant, then added with 1 mL of Trizol reagent to fully lyse other blood cells, and then added with 0.2 mL of trichloride Methane reagent after standing at the room temperature for about 5 minutes. The mixed solution was let stand for around 10 min at the room temperature and centrifuged at 12,000 rpm for another 10 min at 4°C. The supernatant was collected and added isopropanol with the same volume and then centrifuged at the same conditions. After removal of supernatant, the precipitate was air-dried and added with sterilized DEPC water to dissolve. Finally, the concentration, purity, and integrity of the extracted Ribonucleic Acid (RNA) were detected by UV spectrophotometer.

By referring to the PrimeScript™ RT Master Mix (Perfect Real Time) kit from Takara, Japan, reverse transcription of cDNA extracted from RNA was conducted, and the concentration and purity of reverse transcribed cDNA were detected using a UV spectrophotometer. Primers for quantitative detection of NF-*κ*B1, NF-*κ*B2, REL, and *β*-actin genes were synthesized by Shanghai Sangon Bioengineering Co., Ltd.  Table 1 shows the primer information. The reaction system and reaction program were set based on instructions of the TB Green® Premix Ex Taq™ II (Tli RNaseH Plus) kit from Takara Company, Japan, and RT-qPCR amplification was performed using the CFX96 Touch fluorescence quantitative PCR instrument from BIO-RAD Company of the United States. The *β*-actin was undertaken as the internal reference gene, and the NF-*κ*B1, NF-*κ*B2, and REL were detected according to the 2^-△△Ct^ equation.

### 2.8. Statistical Methods

SPSS 19.0 was adopted to organize and analyze collected and detected data. Enumeration data were displayed in frequency (percentage), and the *χ*^2^ test procedure was employed for comparative analysis of differences among or between groups. Measurement data were expressed in the form of mean ± standard deviation ($\bar{x}\pm \text{sd}$), and the independent samples *t*-test procedure was for comparative analysis of differences among or between groups. When *P* > 0.05, the difference was not statistically significant.

## 3. Results

### 3.1. Basic Data of Subjects

Differences in basic data of patients in different groups were collected and compared. It can be known from Table 2 no obvious difference in basic conditions such as gender ratio, age, BMI, hypertension history, diabetes history, and smoking history among of them (*P* > 0.05). There were more patients with systolic blood pressure (SBP), diastolic blood pressure (DBP), and drinking history in the UAP group and SAP group (*P* < 0.05).

### 3.2. Comparison of Blood Routine Indicators of Patients

**Figure 1:**
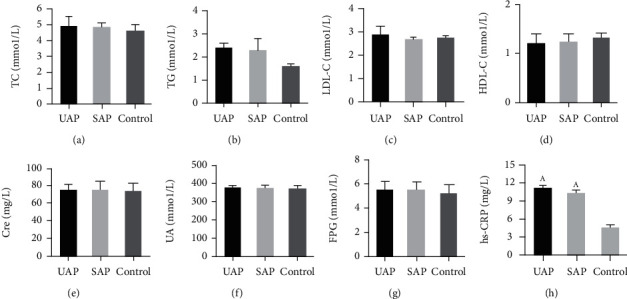
Comparison on blood routine indicators of patients in different groups. (a–h) The comparisons on TC, TG, LDL-C, HDL-C, Cre, UA, and hs-CRP, respectively, A suggested *P* < 0.05 to the values of controls.

The differences in blood routine indicators (TC, TG, LDL-C, HDL-C, Cre, UA, FPG, and hs-CRP) of patients among three groups were compared, as illustrated in Figure 1. As it revealed, no visible difference in the contents of TC, TG, LDL-C, HDL-C, Cre, UA, and FPG of patients was found in the three groups (*P* > 0.05). The hs-CRP results of UAP group, SAP group, and control group were 11.12 ± 1.5 mg/L, 10.23 ± 1.3 mg/L, and 4.51 ± 1.1 mg/L, respectively. The hs-CRP in the UAP and SAP groups showed no obvious difference (*P* > 0.05) but were higher than controls, showing statistically great differences (*P* < 0.05).

### 3.3. Comparison on Visfatin, sVCAM-1, and sICAM-1 Levels

**Figure 2:**
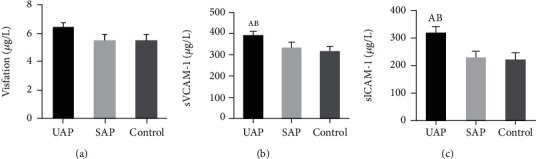
Comparison on Visfatin, sVCAM-1, and sICAM-1 levels. (a–c) The comparisons on Visfatin, sVCAM-1, and sICAM-1 levels, respectively. A and B suggested the difference was obvious to the control group and SAP group, respectively (*P* < 0.05).

Differences in levels of Visfatin, sVCAM-1, and sICAM-1 of patients were compared.  Figure 2 demonstrates that no remarkable difference was detected in the three indicators between SAP patients and healthy subjects (*P* > 0.05), while those of UAP patients were the highest with statistically obvious differences (*P* < 0.05). The Visfatin values of UAP group, SAP group, and control group were 6.43 ± 2.1 *μ*g/L, 5.49 ± 1.6 *μ*g/L, and 5.5 ± 1.4 *μ*g/L, respectively. The sVCAM-1 values of the UAP group, the SAP group, and the control group were 391.6 ± 8.1 *μ*g/L, 334.1 ± 6.8 *μ*g/L, and 315.7 ± 8.9 *μ*g/L, respectively. The values of sICAM-1 in the UAP group, the SAP group, and the control group were 320.8 ± 9.1 *μ*g/L, 230.4 ± 5.8 *μ*g/L, and 222.6 ± 5.6 *μ*g/L, respectively. Figures 2(a)–2(c) illustrate the comparisons on Visfatin, sVCAM-1, and sICAM-1 levels, respectively.

### 3.4. Comparison on Levels of CD63 and CD62P

**Figure 3:**
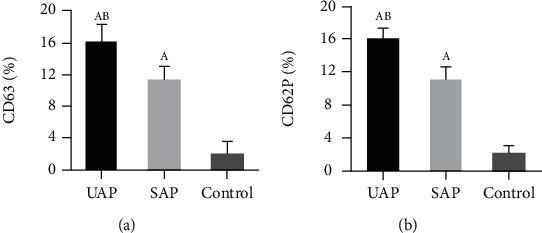
Comparison on levels of CD63 and CD62P. (a, b) The comparison of CD63 and CD62P, respectively. A and B suggested the difference was obvious to the control group and SAP group, respectively (*P* < 0.05).

The differences in serum CD63 and CD62P levels of subjects were compared and shown in Figure 3 below. Figures 3(a) and 3(b) demonstrate the comparisons of CD63 and CD62P of patients in various groups, respectively. The subjects in the UAP group and the SAP group showed higher CD63 and CD62P than the control group (*P* < 0.05); the CD62P results of UAP group, SAP group, and control group were 16.07 ± 2.5%, 11.09 ± 1.8%, and 22.15 ± 0.4%, respectively, while CD63 and CD62P levels in the UAP group were higher compared with the SAP group, with remarkable differences (*P* < 0.05).

### 3.5. Comparison of Serum Inflammatory Factor Levels in Patients

**Figure 4:**
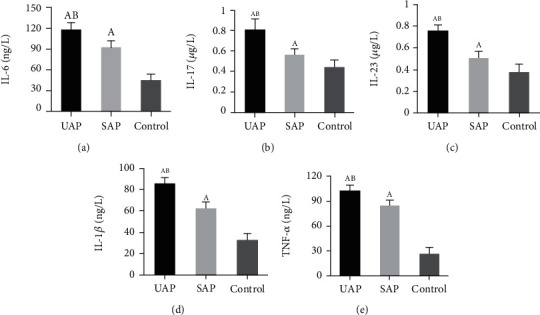
Comparison of serum inflammatory factor levels in patients. (a–e) The comparison of IL-6, IL-17, IL-23, IL-1*β*, and TNF-*α*, respectively. A and B suggested the difference was obvious to the control group and SAP group, respectively (*P* < 0.05).

Levels of inflammatory factors (IL-6, IL-17, IL-23, IL-1*β*, and TNF-*α*) in serum were compared for patients in all groups. The results given in Figure 4 demonstrated that the levels of all above inflammatory factors in the UAP group and the SAP group were higher, with great statistically differences (*P* < 0.05); the levels of UAP patients were the highest (*P* < 0.05).

### 3.6. Comparison of NF-*κ*B1, NF-*κ*B2, and REL mRNA Levels

**Figure 5:**
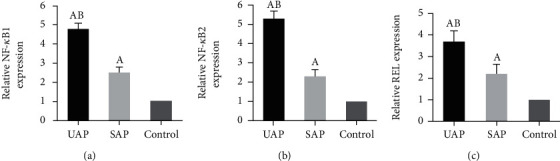
Comparison of NF-*κ*B1, NF-*κ*B2, and REL mRNA levels. (a–c) Comparison of NF-*κ*B1, NF-*κ*B2, and REL mRNA levels, respectively. A and B suggested the difference was obvious to the control group and SAP group, respectively (*P* < 0.05).

The NF-*κ*B1, NF-*κ*B2, and REL mRNA were compared to analyze the differences. As given in Figure 5, the above indicators showed higher levels in UAP patients and SAP patients, showing statistically notable differences (*P* < 0.05); the relative REL expression results of UAP group, SAP group, and control group were 3.77 ± 1.5, 2.2 ± 0.6, and 1 ± 0.4, respectively; the levels of above indicators in the UAP patients were the highest (*P* < 0.05).

## 4. Discussion

CHD is a type of progressive inflammatory disease involving multiple immune cells and inflammatory factors. At present, increasing research evidence shows that systemic inflammatory response exerts a crucial role in the formation and growth of coronary atherosclerosis, and inflammatory response can promote plaque rupture or ulceration [13, 14]. The rupture of coronary atherosclerotic plaque and other factors lead to secondary thrombosis, which can cause complete or incomplete coronary occlusion in patients, clinically manifesting as UAP, AMI, or sudden death [15]. Inflammatory response is an important feature of unstable coronary atherosclerosis, so the detection of inflammatory markers is greatly conductive to the early diagnosis of coronary heart disease [16]. In addition, the specific mechanism of NF-*κ*B-mediated inflammatory response and sVCAM-1 in the process of CHD has not been fully elucidated. To this end, UAP and SAP patients were undertaken as the research subjects in this work to compare and analyze the content changes of inflammatory markers in their serums.

This work showed that Visfatin, sVCAM-1, and sICAM-1 in UAP patients were highly expressed compared with SAP patients and healthy people, but there was no visible difference in the levels of each indicator between SAP patients and healthy people. Visfatin is a cytokine secreted by adipocytes, which can catalyze the rate-limiting enzyme of nicotinamide biosynthesis of nicotinamide adenine dinucleotide, which is important in cell biological process, inflammatory response, and malignant transformation of cells [17]. Studies have confirmed that Visfatin is highly expressed in unstable coronary plaques [18]. sVCAM-1 and sICAM-1 are immunoglobulin superfamily members in peripheral blood, which participate in and mediate the vascular endothelial inflammatory injury response caused by adherent vascular endothelial cells. Therefore, they can be used to evaluate the degree of vascular endothelial inflammatory damage. sVCAM-1 and sICAM-1 are expressed in vascular endothelial cells and are the initiating factors leading to coronary arteriosclerosis, which can be used as evaluation markers for vascular endothelial injury [19, 20]. The above results indicate that Visfatin, sVCAM-1, and sICAM-1 are closely related to vascular endothelial injury and plaque instability in patients with CHD. In addition, it was found in this work that the UAP patients showed higher CD63 and CD62P levels. The activation and degree of activation of platelets can be referred to evaluate the thrombosis tendency of platelets and the accompanying inflammatory response in patients [21]. CD63 and CD62P levels in serum in UAP patients were elevated, indicating that UAP patients showed a highly activated state of platelets, which increased the risk of thrombosis.

Previous studies have confirmed that Visfatin in umbilical vein endothelial cells of human beings can induce endothelial dysfunction by activating NF-*κ*B and inducing the sVCAM-1 and sICAM-1 [22]. NF-*κ*B is a widespread transcriptional regulator in mammalian cells, which participates in regulating the expression of cytokines, biological factors, and chemokines and involves in biological processes such as inflammatory immune response, apoptosis, and transcription [23]. Studies proved that activating the NF-*κ*B pathway is the main pathogenesis of inflammatory response leading to coronary microembolism [24], can enhance the transcription of IL-1*β* and TNF-*α*, and can affect their production, ultimately affecting the process of cardiac dysfunction [25]. Similar to the results of this study, the intervention of clopidogrel in unstable angina pectoris can significantly improve the blood lipid level, reduce the expression of TNF-*α*, IL-1, and IL-6, and downregulate the activity of NF-*κ*B. So it has definite efficacy and good safety and is worthy of clinical promotion [26]. This work revealed that NF-*κ*B1, NF-*κ*B2, and REL mRNA in serum of UAP patients were sharply increased, and it was the same case for the levels of IL-1*β* and TNF-*α* in SAP patients and healthy controls. IL-1*β* and TNF-*α* can stimulate the activation of NF-*κ*B. IL-23 can activate NF-*κ*B under abnormal inflammatory conditions and stimulate T lymphocytes to secrete inflammatory factors after differentiation [27]. In addition, the results of this work revealed that IL-6, IL-17, and IL-23 in UAP patients were also elevated obviously compared with the SAP patients and healthy controls. It suggests that the NF-*κ*B is involved in the activation of the IL-12/IL-17 inflammatory axis and causes an inflammatory response, and UAP may regulate NF-*κ*B activation through a positive feedback pathway.

## 5. Conclusion

This work is aimed at exploring the mechanism of NF-*κ*B pathway-mediated inflammatory response and vascular endothelial cytokines in UAP patients. The results showed that UAP patients had marked activation of the IL-23/IL-17 inflammatory axis, high expression of sVCAM-1 and sICAM-1, and activation of the NF-*κ*B pathway. However, clinical samples were selected to compare and analyze the differences in serum-related indicators between UAP and SAP patients only. In the future, cellular models or animal models would be constructed to explore the specific mechanism of NF-*κ*B pathway activation during UAP. The findings may lay the foundation for early diagnosis of coronary heart disease, improve patient outcomes, and reduce UAP morbidity and mortality.

## Figures and Tables

**Table 1:** Quantitative primer information of target gene.

Gene	Primer sequence (5′→3′)		Product size
NF-*κ*B1	Upstream	GAAGCACGAATGACAGAGGC	137
	Downstream	GCTTGGCGGATTAGCTCTTTT	
NF-*κ*B2	Upstream	AGAGGCTTCCGATTTCGATATGG	89
	Downstream	GGATAGGTCTTTCGGCCCTTC	
REL	Upstream	ACATGGTAATTTGACGACTGCT	204
	Downstream	GCTTCCCAATCGTTCAACACA	
*β*-Actin	Upstream	AAAACTCCCACGGAATCCTCA	216
	Downstream	AATACCGTTGTTCCTTCCCCT	

**Table 2:** Basic data of patients in three groups.

Item	UAP group	SAP group	Control group
Sample size	60	60	30
Gender (males/females)	38/22	40/20	13/17
Age (years old)	62.88 ± 4.13	63.07 ± 3.91	61.59 ± 3.28
BMI (kg/m^2^)	24.21 ± 1.18	24.33 ± 1.09	23.67 ± 2.32
SBP (mmHg)	133.71 ± 16.52^a^	134.05 ± 14.36^a^	123.40 ± 12.83
DBP (mmHg)	85.02 ± 9.17^a^	84.87 ± 8.23^a^	77.95 ± 7.44
Hypertension history (*n* (%))	33 (55.0)	31 (51.7)	14 (46.7)
Diabetes history (*n* (%))	12 (20.0)	10 (16.7)	6 (20.0)
Smoking history (*n* (%))	19 (31.7)	17 (28.3)	8 (26.7)
Drinking history (*n* (%))	28 (46.7)^a^	23 (38.3)^a^	6 (20.0)

Note: a meant *P* < 0.05 compared to the control group.

## Data Availability

The data used to support the findings of this study are included within the article.
